# The accuracy of extended-spectrum beta-lactamase detection in *Escherichia coli* and *Klebsiella pneumoniae* in South African laboratories using the Vitek 2 Gram-negative susceptibility card AST-N255

**DOI:** 10.4102/sajid.v34i1.114

**Published:** 2019-08-07

**Authors:** Andrea L. Young, Mark P. Nicol, Clinton Moodley, Colleen M. Bamford

**Affiliations:** 1 Division of Medical Microbiology, Department of Pathology, Faculty of Health Sciences, University of Cape Town, Cape Town, South Africa; 2 National Health Laboratory Service, Groote Schuur Hospital, Cape Town, South Africa

**Keywords:** Antimicrobial susceptibility testing, Extended-spectrum beta-lactamase (ESBL) Detection, Automated systems for ESBL Detection, Vitek 2 ESBL detection, Gram-negative susceptibility card AST-N255

## Abstract

**Background:**

Phenotypic detection of extended-spectrum beta-lactamases (ESBLs) is based on the inhibition of ESBL enzymes by β-lactamase inhibitors and on the comparison of cephalosporin activity with or without a β-lactamase inhibitor. Many South African diagnostic laboratories rely on the Vitek 2 for automated susceptibility testing and for ESBL detection. However, the Gram-negative susceptibility card currently used locally (AST-N255) has been modified and its accuracy for ESBL detection is not known.

**Methods:**

We randomly selected 50 isolates of *Klebsiella pneumoniae* and *Escherichia coli* from a collection of clinical bloodstream isolates from Groote Schuur Hospital from 2015 to 2016, including ESBL-producing and non-ESBL-producing strains. We used standardised phenotypic (disc diffusion and broth microdilution) and genotypic (conventional polymerase chain reaction (PCR) for *bla_CTX-M_, bla_SHV_* and *bla_TEM_*) methods for detection of ESBLs. We compared ESBL detection by Vitek 2 to a composite reference standard comprising ESBL detection either by both phenotypic methods or by one phenotypic method together with genotypic detection.

**Results:**

The sensitivity of Vitek 2 system for detection of ESBLs was 33/36 or 92% (78% – 97%) for *E. coli*, and 40/40 or 100% (91% – 100%) for *K. pneumoniae*, whilst specificity was 10/10 or 100% (72% – 100%) and 9/10 or 90% (60% – 98%), respectively. This is comparable with previous studies.

**Conclusion:**

Using a composite reference standard of the phenotypic and genotypic methods employed in this study, no Vitek-categorised ESBL *E. coli* or *K. pneumoniae* was found to be a non-ESBL with the exception of possible misinterpretation with *K. pneumoniae* SHV-hyper-producing isolates.

## Background

Extended-spectrum β-lactamases (ESBLs) are enzymes that hydrolyse most penicillins and cephalosporins, including oxyimino-β-lactam compounds, but not cephamycins or carbapenems.^1^ Extended-spectrum β-lactamases are reported to be widespread in South Africa, particularly in *Klebsiella pneumoniae*^2,3^ and in nosocomial infections.^4^ Rates of ESBL-producing *Escherichia coli* are also increasing.^5^

As many ESBL-producing organisms are also resistant to other unrelated antibiotics, such as fluoroquinolones, aminoglycosides and trimethoprim-sulfamethoxazole, high rates of ESBL infections result in increased use of carbapenems, which, in turn, selects for carbapenem-resistant organisms, for which there are few therapeutic options.

In routine diagnostic laboratories, phenotypic methods for the detection of ESBLs are based on the inhibition of ESBL enzymes by β-lactamase inhibitors and on the comparison of cephalosporin activity with or without a β-lactamase inhibitor.^6,7,8^ This principle underlies both agar-based testing and broth-based testing, including automated susceptibility testing systems such as the Vitek 2 (BioMerieux, Marcy-l’Étoile, France) which is widely used in South Africa.

However, various modifications to the Gram-negative susceptibility card to meet local needs have necessitated the removal of the cephalosporin–clavulanic acid combination wells. Consequently, ESBL detection in South African laboratories is now based solely on the pattern of susceptibility and resistance to different cephalosporins. Experience with similarly modified Vitek 2 Gram-negative susceptibility cards elsewhere suggests that the specificity of ESBL detection may be reduced.^9,10^ Whilst the Vitek 2 Advanced Expert System^TM^ (AES), which automatically compares minimum inhibitory concentrations (MICs) of antibiotics to a large database and suggests possible mechanisms of resistance, tends to favour the most conservative options to ensure safe patient treatment, it may thereby add to the over-calling of ESBLs.

The aim of this study was therefore to determine the sensitivity and specificity of the Vitek 2 AST-N255 Gram-negative susceptibility card for the detection of ESBLs in *K. pneumoniae* and *E. coli* when compared to standardised phenotypic and genotypic methods.

## Methods

### Bacterial isolates

The Groote Schuur NHLS microbiology laboratory serves the southern part of the greater Cape Town area with a catchment population of approximately 2 million people. The laboratory receives approximately 3600 blood culture samples a month and from these maintains a stored collection of selected organisms. From the 2015–2016 collection of bloodstream isolates, we randomly selected 50 isolates of *K. pneumoniae* and *E. coli*, including 40 ESBL-producing and 10 non-ESBL-producing strains of each organism. This categorisation was based on the original identification and susceptibility testing (with Vitek 2 AST-N255 Gram-negative susceptibility card) that was reported in routine diagnostic testing. An isolate was considered an ESBL producer if the phenotypic interpretation by the AES of the Vitek 2 included ESBL with or without decreased outer membrane permeability and not an ESBL if only wild type or β-lactamases other than ESBLs were suggested by AES. For rapid identification of blood culture isolates, the laboratory uses a previously validated method of direct inoculation from a concentrated suspension of the bottle fluid.^11^ Isolates had been stored as glycerol stocks at -80 °C and were subcultured and re-tested with the Vitek 2 to confirm identification and susceptibility test results. The same inoculum was used for concurrent phenotypic and genotypic testing as described below. This second Vitek 2 result was considered the definitive result for comparison purposes. Control strains for ESBL detection, as described below, were tested concurrently.

### Phenotypic extended-spectrum beta-lactamase detection

Extended-spectrum β-lactamase production was detected by disc diffusion and broth microdilution methods, performed and interpreted according to CLSI criteria.^6^ Appropriate quality control organisms, namely *E. coli* ATCC ^(R)^ 25922 and *K. pneumoniae* ATCC ^(R)^ 700603, were included for each method in each run.

Disc diffusion testing was performed using the Kirby–Bauer method. Cefotaxime and ceftazidime discs with or without clavulanic acid were utilised, with an increase of ≥ 5 mm in zone inhibition diameter for either cephalosporin in the presence of the inhibitor, indicating the presence of an ESBL.

Broth microdilution was performed using the Sensititre™ ESBL plate format (Trek Diagnostic systems, ThermoScientific Waltham, MA, USA) in accordance with the manufacturer’s instructions. The Sensititre™ ESBL plate includes wells containing cefotaxime and ceftazidime with or without clavulanic acid. A ≥ 3 twofold concentration decrease in MIC for either cephalosporin in the presence of the inhibitor indicates the presence of an ESBL.

The ranges of MICs (in µg/mL) that can be determined using the Sensititre ESBL plate are ≤ 0.25–> 64, ≤ 0.12/4-–>64/4, ≤ 0.25–> 128 and ≤ 0.12/4–> 128/4 for cefotaxime, cefotaxime + clavulanic acid, ceftazidime and ceftazidime + clavulanic acid, respectively. An indeterminate result was reported when it was not possible to calculate the ratio accurately at the limits of MIC range, for example if cefotaxime MIC ≤ 0.25, and cefotaxime + clavulanic acid MIC ≤ 0.12/4. If an indeterminate result was obtained for either cefotaxime or ceftazidime, the isolate was categorised according to the result of the other antibiotic. If indeterminate results were obtained for both antibiotics, the ESBL status was determined by the MICs of the antibiotics, that is, if both cefotaxime and ceftazidime MICs were at the lower limit of the MIC range, the isolate was reported as ESBL negative, whereas if both MICs were at the upper limit of the range, the isolate was reported as ESBL-positive.

### Genotypic detection of extended-spectrum beta-lactamases

Bacterial DNA was extracted from colonies grown on 2% blood agar using the QiaSymphony SP automated extraction platform with the QiaSymphony DSP Virus/Bacteria mini kit, according to the manufacturer’s instructions (Qiagen, Hilden, Germany). To test for the presence of the most commonly occurring ESBL genes *bla*_CTX-M_, *bla*_SHV_ and *bla*_TEM_, conventional PCR assays using primers designed to target internal fragments of these genes were performed. Details of the primer sequences, expected amplicon sizes and amplification conditions are given in the Appendix. The previously described positive and negative controls were included in each run. Selected amplicons including any discrepant genotypic-phenotypic results were submitted for DNA sequencing (Inqaba Biotech, Muckleneuk, Pretoria, South Africa) and the data analysed to confirm the gene identity and genotype, where possible.

### Statistical analysis

The sensitivity and specificity of the Vitek 2 AST-N255 Gram-negative susceptibility card for ESBL detection was compared to a composite reference standard, in which an isolate was defined as an ESBL if either an ESBL was detected by both phenotypic methods, that is, by disc diffusion and by broth microdilution, or if an ESBL was detected by either phenotypic method as well as genotypically. The 95% confidence intervals for proportions were calculated according to Newcombe method.^12^

## Ethical consideration

Ethical clearance was obtained from the Faculty of Health Sciences Human Research Ethics Committee (HREC) (HREC REF: 909/2015).

## Results

We tested 96 isolates including 46 *E. coli* and 50 *K. pneumoniae* isolates. Of the 46 *E. coli* isolates, 33 were ESBL producers and 13 non-ESBL producers, according to the definitive Vitek 2 test results, whilst among the *K. pneumoniae* isolates tested, there were 41 ESBL producers and 9 non-ESBL producers.

### Genotypic detection of extended-spectrum beta-lactamase genes *bla*_CTX-M_, *bla*_SHV_ and *bla*_TEM_

Among the 46 *E. coli* isolates tested, *bla*_CTX-M_ and *bla*_TEM_ were detected in 23 and 13 isolates, respectively. Five isolates contained both *bla*_CTX-M_ and *bla*_TEM_, whilst *bla*_SHV_ was not detected in any isolate. Among the 50 *K. pneumoniae* isolates tested, *bla*_SHV_ was detected in all, with 11 isolates containing no other *bla* gene. The remaining 39 isolates all contained *bla*_CTX-M_ and 27 also contained *bla*_TEM_.

Sequence analysis of a limited number of gene products confirmed the identification of the ESBL-encoding gene *bla*_CTX-M-15_ in four *bla*_CTX-M_ amplicons in *E. coli* and in two *bla*_CTX-M_ amplicons in *K. pneumoniae*. All *bla*_SHV_ and *bla*_TEM_ amplicons sequenced were identified as narrow-spectrum beta-lactamases in both *E. coli* (7 *bla*_TEM-1_ isolates) and in *K. pneumoniae* (5 *bla*_SHv-1,_ 1 *bla*_LEN-17_, 1 *bla*_LEN-19_ and 1 *bla*_TEM-1_). No other amplicons were sequenced.

### Phenotypic detection of extended-spectrum beta-lactamases

All isolates were tested for ESBL production using both phenotypic methods. Using the Sensititre method, an indeterminate result was obtained with both cefotaxime and ceftazidime for 10 *E. coli* and 10 *K. pneumoniae* isolates. These isolates were reclassified according to the MICs of both antibiotics.

### Extended-spectrum beta-lactamase detection by Vitek 2 compared to composite reference method

The composite reference standard comprised either ESBL detection by both phenotypic methods or by a combination of one phenotypic method together with genotypic detection. Given the detection of non-ESBL genes in *bla*_SHV_ and *bla*_TEM_ amplicons, *bla*_CTX-M_ was the sole target included for genotypic ESBL detection.

The sensitivity of Vitek 2 system for detection of ESBLs as compared to the composite reference standard was 33/36 or 92% (78% – 97%) for *E. coli* and 40/40 or 100% (91% – 100%) for *K. pneumoniae*, whilst specificity was 10/10 or 100% (72% – 100%) and 9/10 or 90% (60% – 98%), respectively (see Tables 1 and 2).

**TABLE 1:** Extended-spectrum beta-lactamase detection by Vitek 2 compared to composite reference method in *Escherichia coli*.

Variable	ESBL classification according to composite reference standard		
	Positive	Negative	Total
**Definitive Vitek classification**			
Positive	33	0	33
Negative	3	10	13
**Total**	**36**	**10**	**46**

ESBL, extended-spectrum beta-lactamase.

**TABLE 2:** Extended-spectrum beta-lactamase detection by Vitek 2 compared to composite reference method in *Klebsiella pneumoniae*.

Variable	ESBL classification according to composite reference standard		
	Positive	Negative	Total
**Definitive Vitek classification**			
Positive	40	1	41
Negative	0	9	9
**Total**	**40**	**10**	**50**

ESBL, extended-spectrum beta-lactamase.

Detailed analysis of three *E. coli* and one *K. pneumoniae* isolates that were misclassified by the Vitek 2 is shown in Appendix Table 1-A1: The only isolate mis-categorised as an ESBL by Vitek 2 was a *K. pneumoniae* isolate-resistant to ceftazidime only and lacking inhibition by clavulanic acid. The Vitek phenotype listed SHV hyper-production as an alternative resistance phenotype alongside the ESBL resistance phenotype and *bla*_SHV_ was the sole *bla* gene detected.

Three *E. coli* isolates classified by the Vitek 2 as having acquired penicilllinases were classified as ESBLs according to the composite reference standard, based on the detection of *bla*_CTX-M_ plus phenotypic detection of an ESBL by one method. The antibiotic susceptibility profile of these isolates according to Vitek 2 showed non-susceptibility to ampicillin, co-amoxiclav and cefuroxime and susceptibility to cefotaxime and ceftazidime. The three isolates were also generally susceptible to cefotaxime and ceftazidime by both phenotypic methods with some inconsistent exceptions.

## Discussion

The development of automated susceptibility testing systems and their subsequent introduction into routine diagnostic laboratories sparked an interest in the performance of such systems for the detection of ESBLs and a number of studies on the Vitek 2 were conducted, mostly prior to 2010.^9,10,13,14,15,16,17,18,19^ These studies vary in many key aspects including the use of different Vitek AST cards, which may^10,15,16,17,19^ or may not^9,10,13,14,18^ contain ESBL confirmatory wells with combinations of cephalosporins and clavulanic acid. Different versions of the Vitek AES software were also used, especially in some of the earlier studies.^10,15^ The organisms tested varied with some studies restricted to *E. coli* and *Klebsiella*^13,15,17,18^ as these were the organisms for which phenotypic ESBL testing was recommended by CLSI, whilst others included a wider variety of Enterobacteriaceae including AmpC producers.^9,14,19^ Some studies focussed on clinical isolates,^10,13,14,17,18,19^ whilst others used isolates from large collections specially selected to represent a diversity of resistance mechanisms.^15,16^ Some studies only included isolates presumed to be ESBLs based on screening criteria,^10,18^ whilst the reference methods used for comparison also varied. Overall, sensitivity ranged from 78.0% to 98.1%, whilst specificity showed greater variation, from as low as 33.3% to 99.7%. In general, performance was better with Vitek AST cards containing confirmatory wells,^10,15,16,17,19^ and when restricted to *E. coli* and *Klebsiella*.^10,15,16,17^ In a number of studies, performance was inferior to combination disc testing^9,19^ although other studies suggested that Vitek 2 was adequate for use in routine diagnostic laboratories.^14,16,17,19^ Studies of other automated systems show similar variability in design and limited performance in general.

In this study, the performance of the Vitek 2 AST-N255 card was comparable to previous studies with sensitivities of 92% (78% – 97%) and 100% (91% – 100%) and specificities of 100% (72% – 100%) and 90% (60% – 98%) for *E. coli* and *K. pneumoniae*, respectively, against the composite reference standard. The small number of non-ESBL isolates included in this study meant that confidence intervals for all specificity estimates were relatively wide, ranging between of 60% and 100%.

Limitations of this study included the fact that testing was not performed in duplicate and that the commercial broth microdilution method chosen contained a limited range of antibiotic concentrations which generated many indeterminate results. Genotypic testing was limited to the commonest ESBLs, in particular to the *bla*_CTX-M-1_ family of ESBLs and particularly among *E. coli* other types of ESBLs may not have been detected. Genotypic detection of resistance genes does not necessarily correlate with expression, and this, together with slight technical errors in phenotypic testing, may have resulted in the mis-categorisation of three phenotypically non-ESBL *E. coli* isolates as potential ESBLs. Isolates were selected at random from stored clinical isolates, but as this was a single site study, these isolates may not be representative of resistance genotypes present in other geographic areas. The findings are applicable only to the Vitek AST-N255 card or cards with a similar configuration of cephalosporin and cephalosporin–clavulanic acid combination wells.

However, using a composite reference standard encompassing combinations of the phenotypic and genotypic methods employed in this study, no Vitek-categorised ESBL *E. coli* or *K. pneumoniae* was found to be a non-ESBL with the exception of possible misinterpretation with *K. pneumoniae* SHV-hyper-producing isolates.^20^

## Appendix 1

**TABLE 1-A1:** Analysis of discrepant results for extended-spectrum beta-lactamase detection.

Organism	Isolate	Resistance phenotype and susceptibility profile† according to Vitek 2	ESBL detection by disc diffusion	ESBL detection by Sensititre MIC	Dual phenotypic methods	CTX-M detected by PCR	CTX-M plus either phenotypic method	ESBL detection by composite reference standard
*E. coli*	8	Acquired penicillinase Non-susceptible to ampicillin, co-amoxiclav, cefuroxime Susceptible to cefotaxime, ceftazidime, cefepime, cefoxitin	- Susceptible to cefotaxime and ceftazidime	+§ Susceptible to cefotaxime and ceftazidime	-	+	+	+
*E. coli*	9	Acquired penicillinase Non-susceptible to ampicillin, co-amoxiclav, cefuroxime Susceptible to cefotaxime, ceftazidime, cefepime, cefoxitin	- Susceptible to cefotaxime and ceftazidime	+¶ Susceptible to cefotaxime Non-susceptible to ceftazidime	-	+	+	+
*E. coli*	10	Acquired penicillinase Non-susceptible to ampicillin, co-amoxiclav, cefuroxime Susceptible to cefotaxime, ceftazidime, cefepime, cefoxitin	+‡ Non-susceptible to cefotaxime Susceptible to ceftazidime	+†† Susceptible to cefotaxime and ceftazidime	+	+	+	+
*Klebsiella pneumoniae*	67	ESBL or SHV hyper-producer	-	+	-	-	-	-

† , According to CLSI criteria.

‡ , Cefotaxime zone diameter (mm): cefotaxime + clavulanic acid zone diameter (mm) = 22:32, that is, ESBL-positive; ceftazidime zone diameter (mm): ceftazidime + clavulanic acid zone diameter (mm) = 26:27, that is, ESBL negative.

§ , Cefotaxime MIC (µg/mL): cefotaxime + clavulanic acid MIC (µg/mL) = 1: ≤ 0.12/4, that is, ESBL-positive; ceftazidime MIC (µg/mL): ceftazidime + clavulanic acid MIC (µg/mL) = 0.5: ≤ 0.12/4, that is, indeterminate.

¶ , Cefotaxime MIC (µg/mL): cefotaxime + clavulanic acid MIC (µg/mL) = 0.5: ≤ 0.12/4, that is, indeterminate; ceftazidime MIC (µg/mL): ceftazidime + clavulanic acid MIC (µg/mL) = 16: 0.5/4, that is, ESBL-positive.

†† , Cefotaxime MIC (µg/mL): cefotaxime + clavulanic acid MIC (µg/mL) = 1: ≤ 0.12/4, that is, ESBL-positive; ceftazidime MIC (µg/mL): ceftazidime + clavulanic acid MIC (µg/mL) = 0.5: ≤ 0.12/4, that is, indeterminate.
